# Registration of Fever Nurses

**Published:** 1921-06-25

**Authors:** Claude B. Ker

**Affiliations:** City Hospital, Comiston Road, Edinburgh


*To the Editor of* The Hospital.

### Registration of Fever Nurses.

*To the Editor of* The Hospital.

Sir,—Your courteous note on my letter of last week misses the point which I desired to make but perhaps did not express with sufficient clearness. The *bona-fide* nurses in attendance on the sick will only be admitted to the Register subject to "the conditions under which the knowledge and experience of such nurses has been gained." Was such a limitation imposed on *bona-fide* practitioners of medicine, dentistry, and midwifery? At the present moment, indeed, Sir Watson Cheyne is deploring the proposal to add to the existing Dental Register a large number of unqualified practitioners, and one only has to glance at the penal cases dealt with by the Midwives Board to appreciate the want of training of many registered midwives. Again I ask, why this discrimination against the *bona-fide* "existing" nurse?

I take it that there are many "existing" nurses who have had very insufficient training, but whom the Nursing Council can hardly avoid placing on the general Register. But if one of these women has been foolish enough to take a complete three years' course in a fever hospital and to pass at the end of it what is practically a State examination in the principles of nursing, after a searching preliminary examination in "Anatomy and Physiology," and in "Hygiene and Dietetics" (which, I may add, is of a standard considerably higher than that in most general hospitals), she is not, like the almost untrained nurse, to be allowed a place in the general Register because there happens to be a Fever Register on which it is more convenient to place her! This is an excellent rule for the future, but it seems scarcely fair to apply it to the past.—I am, Sir, yours truly,

Claude B. Ker, M.D.

P.S.—It appears to me that in any case the best security for the general-trained nurse for some years to come will be to join the College of Nursing, which has, at least, a definite standard.

City Hospital, Comiston Road, Edinburgh,
June 18, 1921.

